# Relative contributions of genetic and environmental factors to palatal morphology: a longitudinal twin study

**DOI:** 10.1093/ejo/cjae076

**Published:** 2024-12-20

**Authors:** Jamal Giri, Michelle Bockmann, Alan Brook, Angela Gurr, Lyle Palmer, Matthew Brook O’Donnell, Toby Hughes

**Affiliations:** Adelaide Dental School, Faculty of Health and Medical Sciences, The University of Adelaide, Adelaide, SA 5000, Australia; Adelaide Dental School, Faculty of Health and Medical Sciences, The University of Adelaide, Adelaide, SA 5000, Australia; Adelaide Dental School, Faculty of Health and Medical Sciences, The University of Adelaide, Adelaide, SA 5000, Australia; Adelaide Dental School, Faculty of Health and Medical Sciences, The University of Adelaide, Adelaide, SA 5000, Australia; School of Public Health, Faculty of Health and Medical Sciences, The University of Adelaide, Adelaide, SA 5000, Australia; Australian Institute of Machine Learning, The University of Adelaide, Adelaide, SA 5000, Australia; Annenberg School for Communication, University of Pennsylvania, Philadelphia, PA 19104, United States; Adelaide Dental School, Faculty of Health and Medical Sciences, The University of Adelaide, Adelaide, SA 5000, Australia

**Keywords:** environment, genetics, heritability, palate, twins

## Abstract

**Objectives:**

This study aimed to determine the genetic and environmental contributions to phenotypic variations of palatal morphology during development.

**Methods:**

Longitudinal three-dimensional digital maxillary dental casts of 228 twin pairs (104 monozygotic and 124 dizygotic) at primary, mixed, and permanent dentition stages were included in this study. Landmarks were placed on the casts along the midpoints of the dento-gingival junction on the palatal side of each tooth and the mid-palatine raphe using MeshLab. Palatal widths, depths, length, area, and volume were measured using those landmarks. Univariate genetic structural equation modelling was performed on twin data at each stage of dental development.

**Results:**

Except for anterior depth, all palatal dimensions increased significantly from the primary to permanent dentition stages. The phenotypic variance for most of the palatal dimensions during development was best explained by a model, including additive genetic and non-shared environment variance components. Variance in volume and area in the primary dentition stage was best explained by a model including additive genetic, shared environment, and non-shared environment variance components. For posterior palatal depth and width, narrow-sense heritability estimates were above 0.8 for all dental developmental stages. In contrast, heritability estimates for other palatal traits fluctuated during development.

**Limitation:**

This study was limited to twins of European ancestry.

**Conclusions:**

Additive genetic and non-shared environmental factors primarily influenced palatal morphology during development. While the genetic influence on different aspects of the palate varied throughout development, it was particularly strong in the posterior region of the palate and during the permanent dentition stage.

## Introduction

The palate is a key anatomical structure within the craniofacial complex, essential for the functions of swallowing, speech, and respiration [1]. A strong covariation between palatal shape and the craniofacial complex has been observed in children and adolescents [2]. The palatal morphology interacts with the maxillary arch morphology. The maxillary and mandibular arches are interrelated in width and depth [3], suggesting that the dental arches and the palate are parts of an overall complex adaptive system [4]. The developmental morphology of the palate is an important topic in orthodontic research because alteration of palatal dimensions is often necessary during orthodontic or orthognathic surgical treatments for space management or to correct transverse discrepancy [5]. Understanding the factors that shape the palate during development is important for planning interventions such as palatal expansion.

Variation in palatal morphology between individuals is a complex phenotype under the control of multiple genetic and environmental factors. Non-nutritive sucking behaviours, such as pacifier use, have been associated with changes in palatal shape [6]. Similarly, research has shown that prolonged mouth breathing significantly reduces palatal surface area and volume, further emphasizing the role of environmental factors in shaping palatal morphology [7]. In contrast, other studies have suggested that palatal morphology is strongly influenced by genetics [8–10]. A recent meta-analysis estimated the heritability of palatal depth at 0.56 (95% confidence interval: 0.22–0.90), indicating that both genetic and environmental factors contribute to the observed variance [11]. While both genetic and environmental factors influence the palate, their relative contribution to the overall palatal morphology and its different dimensions during development remains unclear.

Twin studies offer valuable insights into the role of genetic and environmental factors in dentofacial development [12]. The classical twin study design compares monozygotic (MZ) and dizygotic (DZ) twins raised in the same family environment [13]. Monozygotic twins originate from a single fertilized ovum and are considered genetically identical at the nucleotide level, whereas DZ twins arise from two separately fertilized eggs and share on average 50% of their alleles identical by descent. Therefore, the relative contribution of genetic and environmental factors on palatal development can be determined by comparing the degree of phenotypic difference in palatal morphology between MZ twin pairs with those between DZ twin pairs.

Several twin studies have investigated tooth morphology and dental arches [14–18]. However, studies specifically evaluating palatal morphology in twins are limited and mostly restricted to assessing palatal depth and width [8, 9, 19, 20]. Although a recent study evaluated palatal width, depth, surface area, and volume in the permanent dentition among twins, its cross-sectional design limited its ability to evaluate genetic and environmental influences during palate development [21]. In this context, longitudinal twin studies are key to understanding how genetic and environmental factors affect the development of palate as a person grows. A comprehensive review of the literature found no studies examining three-dimensional (3D) changes in palatal morphology from primary to permanent dentition stages in twins.

The objectives of this longitudinal twin study were therefore to:

1. Measure the 3D changes in palatal morphology from primary to permanent dentition stages;
2. Determine the genetic and environmental contributions to phenotypic variance of palatal morphology; and
3. Evaluate temporal changes in the genetic and environmental contributions from primary to permanent dentition stages.

## Materials and methods

### Study samples

Ethics approval was obtained from the Human Research Ethics Committee at the University of Adelaide (Approval number: H-2023-060). The study population was a national cohort of 300 twin pairs recruited from the National Health and Medical Research Council (NHMRC) twin registry. The twins were of European ancestry and did not exhibit craniofacial anomalies. Twin zygosities were confirmed by analysing up to six highly variable genetic loci (FES, vWA31, F13A1, THO1, D21S11, and FGA) genotyped from buccal cell DNA and the accuracy of the process was greater than 99%.

**Figure 1.**
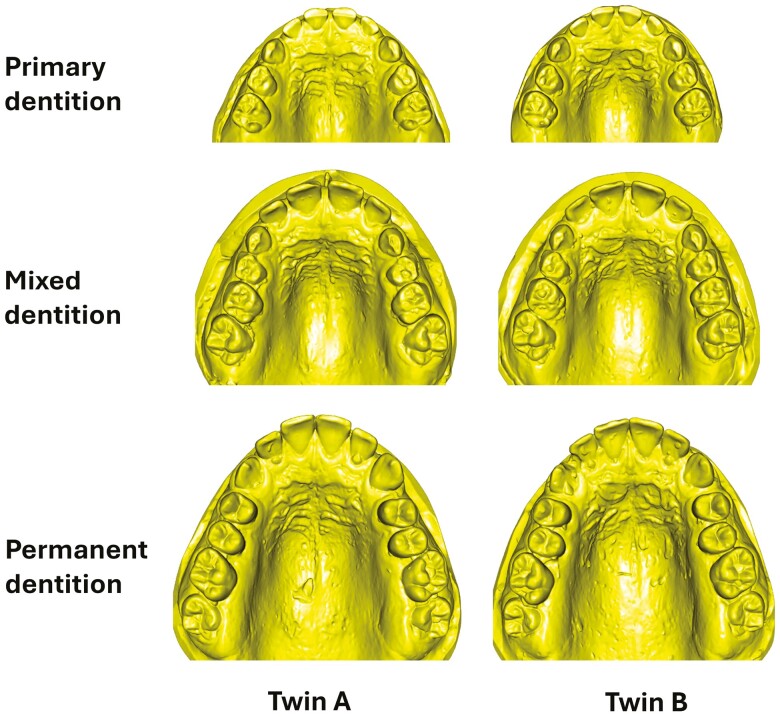
Longitudinal maxillary dental models of twins.

The dental records and associated epidemiological data from twin pairs were collected longitudinally from 1995 to 2006 by the Craniofacial Biology Research Group at the Adelaide Dental School [22]. Dental models were collected at three stages of dental development and comprised the following time points: primary dentition, mixed dentition, and permanent dentition (Figure 1). However, some twins were recruited at the mixed dentition stage and did not have models in the primary dentition stage. Models were created by pouring the alginate impressions of the maxillary and mandibular arches with dental stone.

The stone dental models of the twins were scanned using a benchtop 3D scanner (3Shape, E4, Copenhagen, Denmark) with an accuracy of 4 µm, and were saved in Standard Tessellation Language (.STL) format. The scanned digital models were classified according to dentition stage: (i) primary dentition (all primary teeth from central incisors to second molars), (ii) mixed dentition (primary canines, and first and second primary molars, alongside permanent incisors and first permanent molars), and (iii) permanent dentition (all permanent teeth from central incisors to first permanent molars). Models were excluded based on the following criteria: (a) prior orthodontic treatment; (b) poor quality or damage; and (c) dental anomalies, such as supernumerary teeth or ectopic eruption.

Maxillary digital models from 228 twin pairs (104 MZ and 124 DZ twin pairs) in primary dentition stages were included in the study. In the mixed dentition stage, the sample included 225 twin pairs, and in the permanent dentition stage 170 twin pairs, indicating some attrition from the original sample. An earlier study by Eguchi *et al.* [8] reported intra-class correlation coefficients (ICCs) for palatal depth of 0.84 for MZ twins and 0.42 for DZ twins. An *a priori* power calculation utilizing G*Power (version 3.1.9.7) for *α* = 0.05 and power = 0.90 indicated a minimum sample size of 78 twins was required, consisting of 39 MZ twins and 39 DZ twins, to detect a narrow-sense heritability of 0.77. Our sample size was thus adequate to address our primary hypotheses.

## Palatal measurements

**Figure 2.**
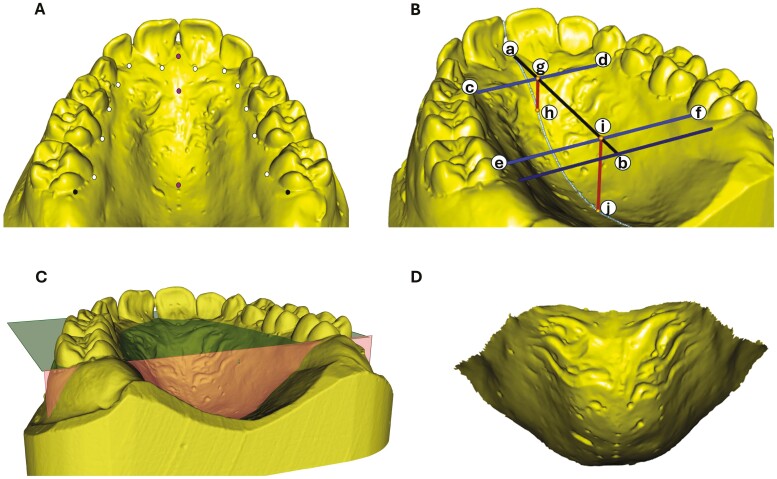
Palatal landmarks and measurements: (A) Landmarks: dento-gingival landmarks (white), mid-palatal landmarks (purple), and posterior landmarks (black); (B) Palatal dimensions: Antero-posterior length (a–b), anterior width (c–d), posterior width (e–f), anterior depth (g–h), and posterior depth (i–j); (C) Gingival plane (green) and posterior plane (red) define the boundary of palate; and (D) cropped section of a palate.

To assess palatal morphology, digital models of the maxillary arch were imported into MeshLab (ISTI-CNR, Pisa, Italy, version 2022.02) for landmark digitization. The midpoints of the dento-gingival junction on the palatal side of each tooth were digitized, along with three landmarks representing the anterior (incisive papilla), middle (midpoint between the right and left canines), and posterior (midpoint between the right and left second molars in primary dentition or the right and left first permanent molars in mixed/permanent dentition) points of the mid-palatal raphe for all maxillary models. A gingival plane was then defined as a singular value decomposition of the dento-gingival junctions on the palatal side of all teeth, accounting for missingness and asymmetry through a process of landmark mirroring and Procrustes transformation [23]. An orthogonal mid-palatal plane was derived in a similar fashion, and subsequently, transverse planes orthogonal to both gingival and mid-palatal planes were derived using relevant gingival antimeres projected to the gingival plane. To mark the posterior limit of the palate, another transverse plane was established using the most distal points on the palatal side of the most posterior teeth (second molars in primary dentition and first permanent molars in mixed and permanent dentitions) (Fig. 2).

Seven palatal measurements were calculated using the assigned landmarks and the defined planes (gingival plane and posterior plane) on each digital model (Table 1). A custom-written R script was used to calculate five linear measurements using standard Euclidean geometry, while a Python script utilizing a trimesh library was used to calculate palatal area and volume.

**Table 1.** Definition of measurements.

Palatal traits	Definition
Anterior width	**Primary and mixed dentitions**: Distance between the midpoints of the dento-gingival junction on the palatal side of the right primary canine and left primary canine. **Permanent dentition**: Distance between the midpoints of the dento-gingival junction on the palatal side of the right permanent canine and left permanent canine.
Posterior width	**Primary dentition**: Distance between the midpoints of the dento-gingival junction on the palatal side of the right primary second molar and left primary second molar. **Mixed and permanent dentitions**: Distance between the midpoints of the dento-gingival junction on the palatal side of the right permanent first molar and left permanent first molar.
Anterior depth	**Primary and mixed dentitions**: Perpendicular distance from the line joining the midpoints of the dento-gingival junctions of primary canines to the deepest point of the palatal vault on the mid-palatal raphae. **Permanent dentition**: Perpendicular distance from the line joining the midpoints of the dento-gingival junctions of permanent canines to the deepest point of the palatal vault on the mid-palatal raphae.
Posterior depth	**Primary dentition**: Perpendicular distance from the line joining the midpoints of the dento-gingival junctions of primary second molars to the deepest point of the palatal vault on the mid-palatal raphae. **Mixed and permanent dentitions**: Perpendicular distance from the line joining the midpoints of the dento-gingival junctions of permanent first molars to the deepest point of the palatal vault on the mid-palatal raphae.
Antero-posterior length	Perpendicular distance from the incisive papilla to the posterior plane of the palate.
Area	Surface area of the palate enclosed by the gingival and posterior planes.
Volume	Volume of the palate enclosed by the gingival and posterior planes.

## Method error

A single examiner (JG) identified the landmarks in all digital models. Identification of the landmarks was repeated for a randomly selected group of 60 models (20 for each of primary, mixed, and permanent dentition) 4 weeks later for intra-examiner reliability. A second investigator (AG) also landmarked the same 60 models for re-calculation to assess the inter-examiner reliability. Systematic error was estimated using the ICC and random error was estimated using the method of the moments estimator (MME) [24].

## Statistical analysis

Mean and standard deviations of the palatal traits were calculated at each stage of dental development. Linear mixed-effects models were performed using the lme4 package to evaluate the association of zygosity, sex, dentition stage, and family ID with continuous palatal dimensions [25]. Zygosity, sex, and dentition stage were modelled as fixed effects, with family ID included as a random effect to account for the clustered nature of twin-pair data. A comparison of the mean palatal dimensions in the mixed dentition stage of twins lost to follow-up and twins followed up in the permanent dentition stage was performed using Student’s *t*-test. Statistical analyses were conducted in R (version 4.3.2), with *P* values <0.05 considered statistically significant.

## Genetic analyses

**Figure 3.**
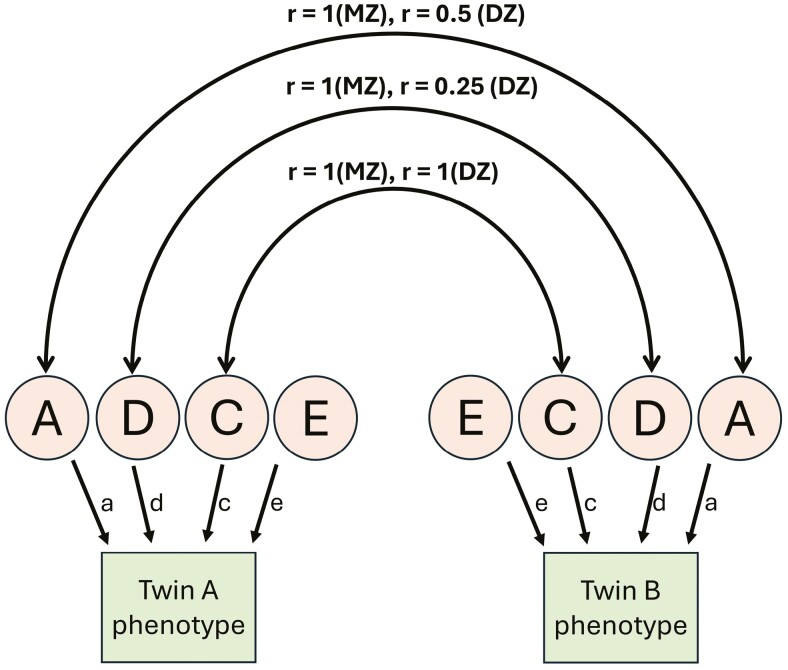
Univariate path diagram depicting four potential influences affecting MZ and DZ twin pairs’ phenotypes: additive genetic factors (A), non-additive genetic factors (D), shared environmental factors (C), and non-shared environmental factors (E). Path coefficients (a, d, c, and e) indicate the relative significance of each influence, while double arrowheads signify correlation (*r*) between latent factors among twin pairs.

Correlation coefficients (ICCs) were calculated for each palatal trait between twin pairs within zygosity groups. A univariate genetic structural equation modelling (SEM) was performed on twin data at each developmental stage to estimate the contributions of genetic and environmental factors to phenotypic variation in palatal morphology across the primary, mixed, and permanent dentitions, using the OpenMx package in R [26] (Fig. 3). The sources of genetic and environmental variations considered in the model fit were:

1. Additive genetic factors (A): genetic factors that cumulatively influence a phenotype.
2. Non-additive genetic factors (D): genetic factors that interact through dominance and epistasis to influence a phenotype.
3. Shared environmental factors (C): environmental factors shared by twins raised in the same family environment that influence a phenotype.
4. Non-shared environmental factors (E): environmental factors specific to each twin that influence a phenotype.

In the genetic model, the expected correlations for additive and non-additive genetic effects are 1 for MZ twins and 0.5 and 0.25, respectively, for DZ twins. For shared environmental effects, the expected correlation is 1 for both MZ and DZ twins raised in the same family, while for non-shared environmental effects, the expected correlation is 0 for both [27]. The genetic analysis was conducted under the assumptions of random mating, equal shared environmental influences for both MZ and DZ twins, and the absence of gene–environment covariation or interaction [28]. ACE and ADE models were fitted to the twin data to estimate the contribution of specific genetic and environmental factors to variations in palatal morphology. As C and D components cannot be estimated simultaneously in twins raised together, ACE and ADE models were fitted separately [29]. Log-likelihoods were computed for each model to evaluate the goodness-of-fit between more complex models and their corresponding sub-models (e.g. ACE vs. AE), using a Chi-square likelihood ratio test. The general approach was to accept a more complex model only if a simpler model showed a significant loss of fit (*P* < 0.05). The parsimony of non-nested models (e.g. CE vs. AE) was assessed using Akaike’s information criteria (AIC) with a smaller value suggesting a better fit. The simplest model that could not be rejected by either the Chi-square likelihood value or AIC was considered the most parsimonious (best-fitting) model. For the most parsimonious model, narrow-sense heritability (*h*^2^) estimates were reported for each palatal trait, calculated as the ratio of additive genetic variation to the total phenotypic variation.

## Results

There was excellent intra-examiner (ICC: 0.97–0.99) and inter-examiner (ICC: 0.95–0.99) reliability for all palatal measurements. Method of the moments estimator showed random errors of less than 1 mm for all linear palatal measurements, 16 mm² for palatal area, and 123 mm³ for palatal volume (Supplementary document 1).

The mean ages of the twins at the different dentition stages were primary 5.8 years (range: 3.5–7 years), mixed 9.4 years (range: 6.8–12.6 years), and permanent 14.3 years (range: 11.6–17.3 years). All palatal dimensions increased significantly from the primary to the permanent dentition stages except anterior depth which did not change significantly. The increment was highest for palatal volume at 134.6% and lowest for anterior depth at 2.4%. No statistically significant differences were observed between MZ and DZ twins except for volume and posterior width in mixed dentition and anterior depth in permanent dentition. When Bonferroni correction was applied to the overall significance level of 0.05, no statistically significant differences were observed between the MZ and DZ twins (Ssupplementary document 2). While males had larger palatal dimensions compared to females, these differences were not always statistically significant. Even when the differences were statistically significant, they were less than 1 mm for all linear measurements except for the antero-posterior length in the permanent dentition (Table 2). As a result, pooled data from male and female twins were used for subsequent genetic analysis. No statistically significant differences existed in the mean palatal dimensions in the mixed dentition between twins lost to follow-up and those followed up in the permanent dentition stage. This suggests that sample attrition did not introduce any bias into the results.

**Table 2.** Mean values of palatal dimensions in different dentition stages.

Palatal dimensions in different dentition stages	Mean (SD)					Overall change from primary dentition
	Overall	Male	Female	MZ	DZ	
Primary	*n* = 456	*n* = 214	*n* = 242	*n* = 208	*n* = 248	
Area (mm^2^)	753.7 (78.4)	770.1 (70.1)	739.9 (82.5)*	756.7 (73.9)	751.1 (82.3)	–
Volume (mm^3^)	2630.1 (471.7)	2681.2 (433.3)	2587.1 (498.7)*	2661.1 (482.8)	2602.3 (461.1)	–
Anterior width (mm)	22.1 (1.9)	22.6 (1.8)	21.8 (1.9)*	22.2 (1.8)	22.1 (2.1)	–
Posterior width (mm)	27.4 (2.1)	27.9 (2.2)	27.0 (1.9)*	27.6 (2.1)	27.3 (2.1)	–
Anterior depth (mm)	4.6 (1.2)	4.4 (1.1)	4.7 (1.3)	4.7 (1.3)	4.5 (1.2)	–
Posterior depth (mm)	10.6 (1.3)	10.6 (1.4)	10.5 (1.3)	10.7 (1.3)	10.5 (1.4)	–
Antero-posterior length (mm)	25.3 (1.6)	25.5 (1.5)	25.2 (1.7)	25.4 (1.6)	25.3 (1.7)	–
**Mixed**	*n* = 450	*n* = 220	*n* = 230	*n* = 212	*n* = 238	
Area (mm^2^)	1240.7 (116.2)	1279.2 (106.9)	1202.9 (112.7)*	1250.4 (120.8)	1231.9 (111.4)	64.6%
Volume (mm^3^)	5338.2 (905.0)	5604.3 (856.2)	5077.3 (877.1)*	5464.6 (949.1)	5224.7 (849.8)*	103.0%
Anterior width (mm)	24.7 (1.9)	25.1 (1.8)	24.4 (2.0)*	24.7 (1.9)	24.8 (1.9)	11.7 %
Posterior width (mm)	31.9 (2.3)	32.3 (2.2)	31.6 (2.3)*	32.3 (2.3)	31.6 (2.2)*	16.5 %
Anterior depth (mm)	4.5 (1.3)	4.5 (1.3)	4.5 (1.4)	4.7 (1.4)	4.3 (1.2)	-1.3 %
Posterior depth (mm)	10.6 (1.7)	10.8 (1.8)	10.4 (1.6)*	10.6 (1.7)	10.6 (1.7)	0.5 %
Antero-posterior length (mm)	35.8 (2.1)	36.1 (1.9)	35.4 (2.1)*	35.9 (2.1)	35.6 (2.0)	41.1 %
**Permanent**	*n* = 340	*n* = 175	*n* = 165	*n* = 154	*n* = 186	
Area (mm^2^)	1266.8 (146.1)	1296.4 (127.1)	1234.0 (158.7)*	1275.2 (160.1)	1259.0 (131.6)	68.1%
Volume (mm^3^)	6169.4 (1121.5)	6387.9 (1103.5)	5927.1 (1094.7)*	6275.1 (1172.8)	6070.3 (1065.3)	134.6%
Anterior width (mm)	23.9 (2.1)	24.3 (1.9)	23.6 (2.1)*	23.8 (2.0)	24.1 (2.1)	8.2%
Posterior width (mm)	33.5 (2.8)	33.9 (2.8)	33.1 (2.6)*	33.6 (2.9)	33.4 (2.6)	22.3%
Anterior depth (mm)	4.7 (1.6)	4.6 (1.5)	4.8 (1.7)	5.0 (1.8)	4.4 (1.4)*	2.4%
Posterior depth (mm)	13.2 (2.1)	13.6 (2.1)	12.8 (1.9)*	13.3 (2.1)	13.1 (2.0)	25.1%
Antero-posterior length (mm)	34.9 (2.3)	35.6 (2.1)	34.2 (2.3)*	34.9 (2.3)	34.9 (2.3)	37.8%

Abbreviations: DZ, Dizygotic; MZ, Monozygotic; SD, Standard deviation.

*P value <0.05 (statistically significant difference between groups), %, percentage, *n*, number of samples.

**Figure 4.**
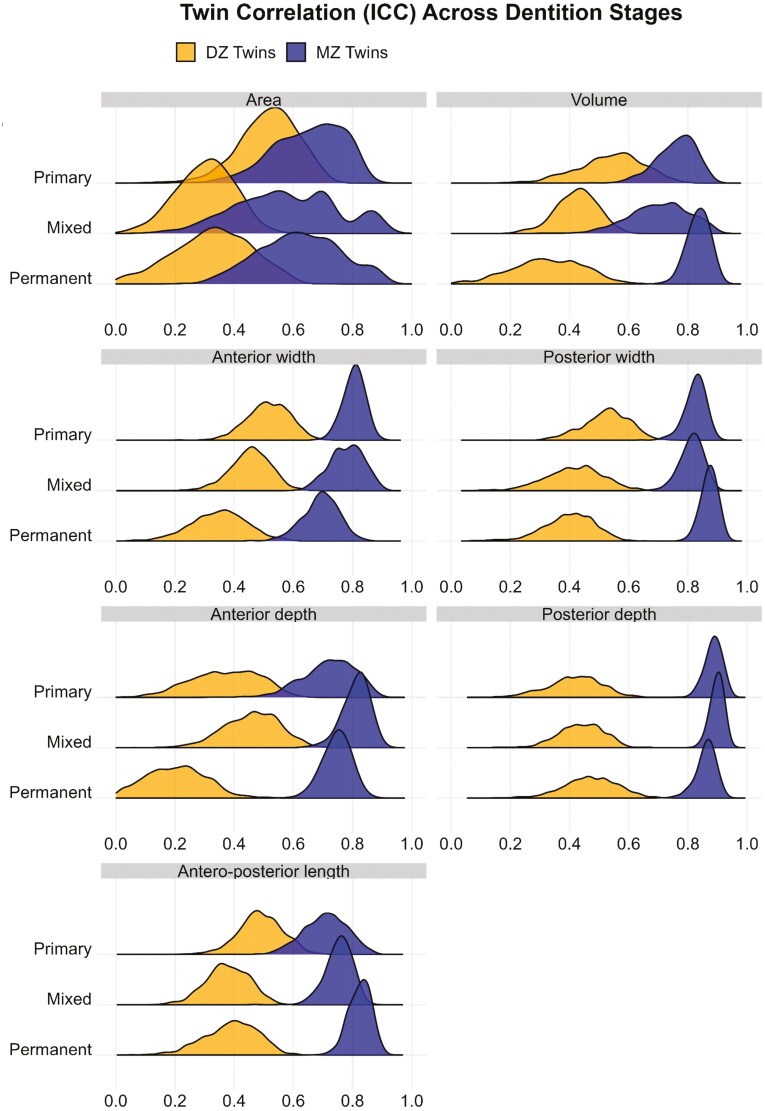
Ridge plots with density curves for ICC distribution.

The ICCs for palatal traits between MZ twins ranged from 0.65 to 0.89 in the primary dentition, 0.58 to 0.90 in the mixed dentition, and 0.62 to 0.87 in the permanent dentition. Among the palatal traits, palatal area exhibited the lowest correlation between MZ twins at each dentition stage. The ICCs of DZ twins were smaller than MZ twins and ranged from 0.37 to 0.54 in the primary dentition, 0.30–0.46 in the mixed dentition, and 0.19–0.47 in the permanent dentition. The ridge plots for the ICCs of MZ and DZ twins showed changes in the distributions between the primary and permanent dentitions with a marked shift observed for palatal volume, posterior width, anterior depth, and antero-posterior length. These changes affected both the means and variances of the distributions as well as their overlap/separation (Fig. 4).

**Figure 5.**
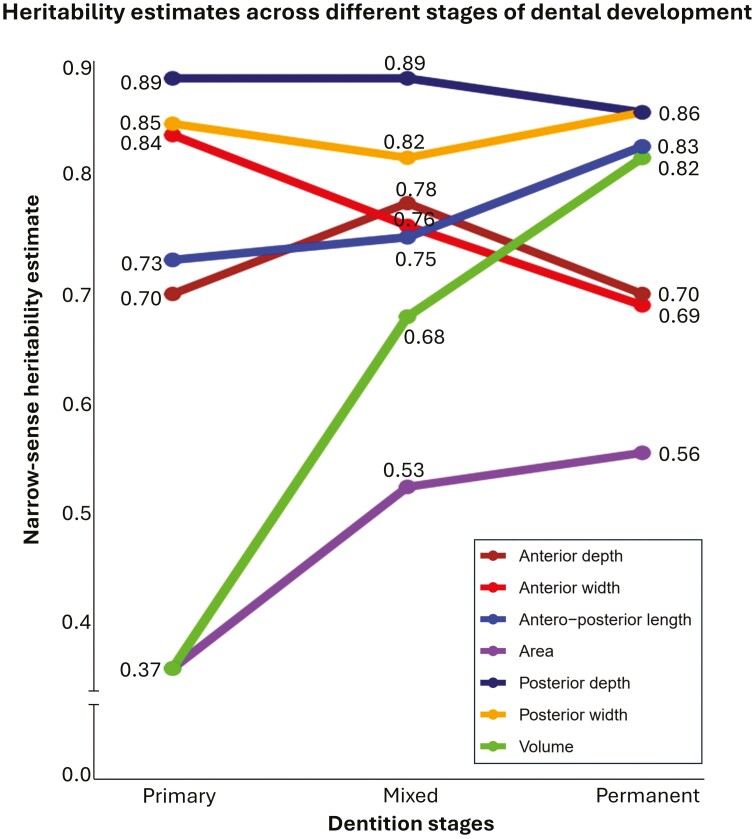
Narrow-sense heritability estimates of palatal traits across different stages of dental development.

The AE model was the most parsimonious for explaining the observed variance for most of the palatal traits in different dentition stages except the palatal area and volume in the primary dentition stage, for which the ACE model demonstrated superior fit (Table 3). Palatal traits exhibited varying narrow-sense heritability estimates across different stages of dental development (Fig. 5). Heritability estimates for posterior width and depth remained consistently high, with values above 0.8 throughout development, whereas estimates for palatal area and volume during the primary dentition stage were both 0.37 and gradually increased thereafter.

**Table 3.** Estimates of variance components and 95% confidence intervals for palatal traits in different stages of dentition.

Palatal traits	Model	AIC	Variance components						
			A	95% CI	C	95% CI	E	95% CI	*h* ^2^
**Primary dentition**									
Area	ACE	3792.5	0.37	0.15–0.51	0.32	0.16–0.43	0.31	0.24–0.41	0.37
Volume	ACE	4968.6	0.37	0.22–0.48	0.38	0.26–0.46	0.25	0.19–0.31	0.37
Anterior width	AE	1272.1	0.84	0.77–0.88	–	–	0.16	0.11–0.23	0.84
Posterior width	AE	1342.7	0.85	0.78–0.89	–	–	0.15	0.11–0.22	0.85
Anterior depth	AE	1039.4	0.70	0.59–0.78	–	–	0.30	0.22–0.40	0.70
Posterior depth	AE	1038.8	0.89	0.84–0.92	–	–	0.11	0.07–0.15	0.89
Antero-posterior length	AE	1243.3	0.73	0.63–0.80	–	–	0.27	0.19–0.36	0.73
**Mixed dentition**									
Area	AE	4586.2	0.53	0.41–0.63	–	–	0.47	0.36–0.58	0.53
Volume	AE	6057.4	0.68	0.60–0.74	–	–	0.32	0.25–0.39	0.68
Anterior width	AE	1490.2	0.76	0.67–0.82	–	–	0.24	0.17–0.32	0.76
Posterior width	AE	1557.8	0.82	0.74–0.86	–	–	0.18	0.13–0.25	0.82
Anterior depth	AE	1177.6	0.78	0.71–0.84	–	–	0.22	0.15–0.28	0.78
Posterior depth	AE	1315.6	0.89	0.85–0.92	–	–	0.11	0.07–0.14	0.89
Antero-posterior length	AE	1508.9	0.75	0.66–0.82	–	–	0.25	0.17–0.33	0.75
**Permanent dentition**									
Area	AE	3562.5	0.56	0.44–0.66	–	–	0.44	0.33–0.55	0.56
Volume	AE	4641.9	0.82	0.76–0.86	–	–	0.18	0.13–0.23	0.82
Anterior width	AE	1150.7	0.69	0.56–0.78	–	–	0.31	0.21–0.43	0.69
Posterior width	AE	1265.8	0.86	0.79–0.90	–	–	0.14	0.09–0.20	0.86
Anterior depth	AE	1023.6	0.70	0.58–0.78	–	–	0.30	0.21–0.41	0.70
Posterior depth	AE	1100.9	0.86	0.79–0.89	–	–	0.14	0.10–0.20	0.86
Antero-posterior length	AE	1191.5	0.83	0.75–0.88	–	–	0.17	0.11–0.24	0.83

Abbreviations: AIC, Akaike’s information criterion score for the best-fitting model; A, additive genetic variance; C, shared environmental variance; E, non-shared environmental variance; CI, confidence interval; *h*^2^, narrow-sense heritability.

## Discussion

Our longitudinal twin study has investigated palate development from the primary to permanent dentition stages in order to determine the relative influence of genetic and environmental factors. An AE model was the most parsimonious for explaining the phenotypic variance of palatal development, except for palatal surface area and volume in the primary dentition stage. Palatal dimensions increased gradually with age, except for the anterior palatal depth which did not change significantly. These dimensions at primary, mixed, and permanent dentition stages may be useful as baseline measurements for comparison in future studies of the growing dental arches. The palatal volume increased by up to 135% and the palatal area by 68%, while anterior palatal depth showed only a modest change of 2% from the primary to the permanent dentition stages. This highlights the varying scaling behaviours of different palatal dimensions, which may explain why the shape of the palate changes during growth and warrants further investigation.

The ICCs showed a greater correlation between MZ twins compared with DZ twins for all palatal traits across the various stages of dental development. For most palatal traits, the ICC between MZ twins was nearly twice those of DZ twins, suggesting an influence of additive genetic factors. For palatal volume, posterior width, anterior depth, and antero-posterior length in the permanent dentition, as well as posterior depth in both the primary and mixed dentition stages, the MZ twin correlations were more than twice those of the DZ twins, suggesting an influence of non-additive genetic factors. However, genetic dominance (non-additive genetic influence) could not be detected during genetic SEM, possibly because the study was underpowered to detect genetic dominance for twins raised together [30]. The marked changes in the ICC distributions for palatal volume, posterior width, anterior depth, and antero-posterior length from the primary to the permanent dentition stages suggest variation in the degree of genetic effect on these parameters throughout this developmental period.

While the AE model was the most parsimonious overall, the ACE model best explained the variation in palatal area and volume in the primary dentition stage, suggesting a shared environmental influence alongside additive genetic and non-shared environmental influence. It could be hypothesized that the shared environmental influence observed for palatal area and volume in the primary dentition stage might be attributed to the use of pacifiers by the twins during their early years. There is evidence that pacifier usage influences palatal morphology [31, 32], but it could not be verified due to the lack of information on pacifier usage among our twin sample. Further research could test this hypothesis by comparing the palatal characteristics of twin pairs who used pacifiers with those who did not.

Our finding that additive genetic and non-shared environmental factors influence palatal traits in the permanent dentition stage aligns with earlier studies [8, 9]. Although Sidlauskiene *et al.* [21] had similar findings, that is, that additive genetic and non-shared environmental factors contributed to the phenotypic variances in posterior width, posterior depth, area, and volume, they also reported that dominant genetic and non-shared environmental factors (DE model) best explained the variance in anterior palatal depth and width, which contrasts with our results. Dominant genetic influence without any additive genetic influence is highly unlikely, and given the relatively small sample size (85 twins) of their study [21], this finding requires further investigation, as D is notoriously difficult to detect in small samples.

Five studies estimated the heritability of posterior palatal depth in the permanent dentition stages, reporting a wide range of values. There were, however, methodological differences in how palatal depth was measured and heritability was estimated. Sharma *et al.* [19] and Lapter *et al.* [20] measured palatal depth from the occlusal surface of the teeth, while Eguchi *et al.* [8], Negishi *et al.* [9], and Sidlauskiene *et al.* [21] used the gingival margin. In addition, Sharma *et al.* [19] and Lapter *et al.* [20] reported broad-sense heritabilities from twin correlations, while the others estimated narrow-sense heritabilities using a genetic structural equation model. The variations in the heritability estimates (0.11–0.86) among these studies could be attributed to methodological differences. In terms of both palatal depth measurement and heritability estimation, our approach was similar to that of Eguchi *et al.* [8], Negishi *et al.* [9], and Sidlauskiene *et al.* [21] and our finding of a strong genetic influence on posterior palatal depth (*h*² = 0.8) is consistent with their results. Furthermore, our findings that the palatal area is moderately influenced by genetics (*h*² = 0.56) and that palatal volume is strongly influenced (*h*² = 0.82) in the permanent dentition stage, align with those of Sidlauskiene *et al.* [21].

Our study is the first to utilize a longitudinal classical twin design to evaluate palate development and demonstrate how genetic and environmental influences change over childhood development. Narrow-sense heritability estimates for posterior palatal width and depth were above 0.8 at all stages of dental development with relatively stable values suggestive of a strong genetic influence on the posterior part of the palate (Fig. 5). The genetic influence on the anterior palate fluctuated during development. For the anterior palatal width, there was a sharp decline in the heritability estimate from 0.84 in primary dentition to 0.69 in permanent dentition, indicative of an increasing environmental influence with age. In contrast, the genetic influence on palatal length increased, with heritability rising from 0.73 in primary dentition to 0.83 in permanent dentition. Heritability estimates for palatal area and volume gradually increased over the transition from primary to permanent dentition, with volume showing a particularly marked rise, suggesting a growing genetic influence. Heritability estimates for all palatal traits in the permanent dentition stage ranged from 0.56 to 0.86, exceeding the average heritability estimate of 0.49 for human traits reported in twin studies over the past 50 years [33]. Overall, the palate was under a strong genetic influence during the permanent dentition stage and this finding has substantial clinical implications.

A comprehensive understanding of dimensional changes, along with genetic and environmental influences on the palate during development, will assist clinicians to optimize the timing of interventions and to set realistic treatment goals. Palatal expansion is a common procedure in orthodontics to correct transverse maxillary and tooth-size arch length discrepancies. The success of this procedure depends on the ossification of the mid-palatal suture which progressively ossifies with age, becoming increasingly rigid and less responsive to orthodontic forces. According to Melson [34], the mid-palatal suture ossifies around the age of 15 in females and 17 in males. Studies have shown that palatal expansion during the permanent dentition stage results in an inverted triangular expansion, with a greater opening in the anterior region compared to the posterior region of the palate, even when the suture has been completely opened [35, 36]. This could be related to the observed strong genetic influence in the posterior palate during the permanent dentition stage, which is often associated with a less favourable prognosis for successful orthodontic outcomes [37]. Since orthodontic force is considered an environmental factor [38], timing palatal expansion during periods when palatal traits have lower heritability could provide a better opportunity to induce desired changes, as the anatomy is more responsive to environmental effects, leading to more predictable outcomes. The findings of this study suggest that a good time for palatal expansion is before the permanent dentition stage. However, it is crucial to understand that heritability is a population-level concept and orthodontic treatment outcome depends on how well an individual responds to a change in environmental factors which can even influence a trait with high heritability [39].

Increased genetic influence on the developing palate as childhood progresses might also explain the relapse following palatal expansion. An average post-treatment relapse of approximately 24% in palatal expansion between ages 13 and 20 years has been reported [40]. The long-term stability of orthodontic treatment outcomes primarily depends on achieving a new balance between genetic and environmental factors [41]. It can be argued that increased genetic influence on the palate during the permanent dentition stage disrupts this balance, leading to post-treatment relapse. While the role of genetics in the aetiology of malocclusion has been investigated, its influence on post-treatment relapse has been largely overlooked. Genetic influence on relapse after orthodontic correction offers a promising new direction for orthodontic research.

Although this study has many strengths, such as its longitudinal design, accurate classification of twin zygosity, and the use of a robust genetic structural equation model to assess the genetic and environmental influences on the palate, it also has some limitations. The inclusion of twins of European ancestry only may limit the generalizability of the findings to other populations. Another limitation was the lack of information on habits such as mouth breathing, digit sucking, or pacifier use, which could have influenced the palatal morphology of twins. In addition, the absence of lateral cephalograms prevented the correlation of palatal development with cervical vertebral maturation stages in these twins. Future research could use multivariate genetic analysis, together with geometric morphometric analysis, to enable the capacity to examine both transitory and cumulative influences of genes and environment on palatal size and shape during development.

## Conclusions

Palatal dimensions increased significantly from the primary to the permanent dentition stages except for anterior depth. Additive genetic and non-shared environmental factors primarily influenced palatal morphology, with a strong genetic influence in the posterior region of the palate. While the genetic influence on different aspects of the palate varied during development, it was particularly strong during the permanent dentition stage. These findings suggest that palatal expansion should be performed before the permanent dentition stage for a more predictable outcome.

## Supplementary Material

cjae076_suppl_Supplementary_Material

## Data Availability

The de-identified maxillary digital models, demographic information of the twins, and measurement data will be made available upon reasonable request to the data custodian (Prof Toby Hughes).
