# Supplementary material for: Relative contributions of genetic and environmental factors to palatal morphology: a longitudinal twin study
Source: Eur J Orthod. 2024 Dec 20;47(1):cjae076. doi: 10.1093/ejo/cjae076 (PMC11659640; doi:10.1093/ejo/cjae076)
Supplement: cjae076_suppl_Supplementary_Material [file cjae076_suppl_supplementary_material.docx]

| **Palatal measurements** | **Intra-examiner ICC** | **Inter-examiner ICC** | **Intra-examiner MME** | **Inter-examiner MME** |
| --- | --- | --- | --- | --- |
| **Area (mm^2^)** | 0.99 | 0.99 | 13.45 | 16.33 |
| **Volume (mm^3^)** | 0.99 | 0.99 | 104.34 | 122.79 |
| **Anterior width (mm)** | 0.99 | 0.95 | 0.35 | 0.22 |
| **Posterior width (mm)** | 0.99 | 0.98 | 0.40 | 0.34 |
| **Anterior depth (mm)** | 0.97 | 0.97 | 0.24 | 0.23 |
| **Posterior depth (mm)** | 0.98 | 0.95 | 0.18 | 0.20 |
| **Antero-posterior length (mm)** | 0.97 | 0.98 | 0.60 | 0.66 |

**Supplementary document 1: Systematic and random errors for palatal measurements**

ICC: Intra-class correlation coefficient; MME: Method of the moments estimator (random error)

| **Palatal dimensions in different dentition stages** | **Mean (SD)** | | | | | **Significance (p-value)** | |
| --- | --- | --- | --- | --- | --- | --- | --- |
|  | **Overall** | **Male** | **Female** | **MZ** | **DZ** | **Male vs. Female** | **MZ vs. DZ** |
| **Primary** | n = 456 | n = 214 | n = 242 | n = 208 | n = 248 |  |  |
| Area (mm^2^) | 753.7 (78.4) | 770.1 (70.1) | 739.9  (82.5)* | 756.7 (73.9) | 751.1 (82.3) | <0.001 | 0.485 |
| Volume (mm^3^) | 2630.1 (471.7) | 2681.2 (433.3) | 2587.1 (498.7) | 2661.1 (482.8) | 2602.3 (461.1) | 0.048 | 0.217 |
| Anterior width (mm) | 22.1 (1.9) | 22.6 (1.8) | 21.8 (1.9)* | 22.2 (1.8) | 22.1 (2.1) | <0.001 | 0.783 |
| Posterior width (mm) | 27.4 (2.1) | 27.9 (2.2) | 27.0 (1.9)* | 27.6 (2.1) | 27.3 (2.1) | <0.001 | 0.213 |
| Anterior depth (mm) | 4.6 (1.2) | 4.4 (1.1) | 4.7 (1.3) | 4.7 (1.3) | 4.5 (1.2) | 0.050 | 0.110 |
| Posterior depth (mm) | 10.6 (1.3) | 10.6 (1.4) | 10.5 (1.3) | 10.7 (1.3) | 10.5 (1.4) | 0.310 | 0.203 |
| Antero-posterior length (mm) | 25.3 (1.6) | 25.5 (1.5) | 25.2 (1.7) | 25.4 (1.6) | 25.3 (1.7) | 0.054 | 0.553 |
| **Mixed** | n = 450 | n = 220 | n = 230 | n = 212 | n = 238 |  |  |
| Area (mm^2^) | 1240.7 (116.2) | 1279.2 (106.9) | 1202.9 (112.7)* | 1250.4 (120.8) | 1231.9 (111.4) | <0.001 | 0.107 |
| Volume (mm^3^) | 5338.2 (905.0) | 5604.3 (856.2) | 5077.3 (877.1)* | 5464.6 (949.1) | 5224.7 (849.8) | <0.001 | 0.007 |
| Anterior width (mm) | 24.7 (1.9) | 25.1 (1.8) | 24.4 (2.0)* | 24.7 (1.9) | 24.8 (1.9) | <0.001 | 0.693 |
| Posterior width (mm) | 31.9 (2.3) | 32.3 (2.2) | 31.6 (2.3) | 32.3 (2.3) | 31.6 (2.2) | 0.002 | 0.006 |
| Anterior depth (mm) | 4.5 (1.3) | 4.5 (1.3) | 4.5 (1.4) | 4.7 (1.4) | 4.3 (1.2) | 0.547 | 0.100 |
| Posterior depth (mm) | 10.6 (1.7) | 10.8 (1.8) | 10.4 (1.6) | 10.6 (1.7) | 10.6 (1.7) | 0.014 | 0.961 |
| Antero-posterior length (mm) | 35.8 (2.1) | 36.1 (1.9) | 35.4 (2.1)* | 35.9 (2.1) | 35.6 (2.0) | <0.001 | 0.251 |
| **Permanent** | n = 340 | n = 175 | n = 165 | n = 154 | n = 186 |  |  |
| Area (mm^2^) | 1266.8 (146.1) | 1296.4 (127.1) | 1234.0 (158.7)* | 1275.2 (160.1) | 1259.0 (131.6) | <0.001 | 0.332 |
| Volume (mm^3^) | 6169.4 (1121.5) | 6387.9 (1103.5) | 5927.1 (1094.7)* | 6275.1 (1172.8) | 6070.3 (1065.3) | <0.001 | 0.108 |
| Anterior width (mm) | 23.9 (2.1) | 24.3 (1.9) | 23.6 (2.1) | 23.8 (2.0) | 24.1 (2.1) | 0.002 | 0.249 |
| Posterior width (mm) | 33.5 (2.8) | 33.9 (2.8) | 33.1 (2.6) | 33.6 (2.9) | 33.4 (2.6) | 0.008 | 0.561 |
| Anterior depth (mm) | 4.7 (1.6) | 4.6 (1.5) | 4.8 (1.7) | 5.0 (1.8) | 4.4 (1.4) | 0.283 | 0.002 |
| Posterior depth (mm) | 13.2 (2.1) | 13.6 (2.1) | 12.8 (1.9)* | 13.3 (2.1) | 13.1 (2.0) | <0.001 | 0.464 |
| Antero-posterior length (mm) | 34.9 (2.3) | 35.6 (2.1) | 34.2 (2.3)* | 34.9 (2.3) | 34.9 (2.3) | <0.001 | 0.947 |

**Supplementary document 2: Comparison of mean values of palatal dimensions in different dentition stages**

MZ: Monozygotic, DZ: Dizygotic, SD: Standard deviation, n = number of samples, *: statistically significant difference at p<0.001 after Bonferroni correction
